# Nordentatin Inhibits Neuroblastoma Cell Proliferation and Migration through Regulation of GSK-3 Pathway

**DOI:** 10.3390/cimb44030070

**Published:** 2022-02-24

**Authors:** Chantana Boonyarat, Panatchakorn Boonput, Nantakorn Tongloh, Rawiwun Kaewamatawong, Suchada Chaiwiwatrakul, Chavi Yenjai, Pornthip Waiwut

**Affiliations:** 1 Faculty of Pharmaceutical Sciences, Khon Kaen University, Khon Kaen 40002, Thailand; chaboo@kku.ac.th; 2 Faculty of pharmaceutical sciences, Ubon Ratchathani University, Ubon Ratchathani 34190, Thailand; panatchakorn.bo.59@ubu.ac.th (P.B.); sunisa.ma.59@ubu.ac.th (N.T.); rawiwun.k@ubu.ac.th (R.K.); 3 Department of English, Faculty of Humanities and Social Sciences, Ubon Ratchathani Rajabhat University, Ubon Ratchathani 34000, Thailand; suchadachai65@gmail.com; 4 Natural Products Research Unit, Department of Chemistry and Center of Excellence for Innovation in Chemistry, Faculty of Science, Khon Kaen University, Khon Kaen 40002, Thailand; chayen@kku.ac.th

**Keywords:** nordentatin, cell proliferation, apoptosis, migration, GSK-3

## Abstract

Cancer is caused by abnormal cell changes leading to uncontrolled cell growth. The specific characteristics of cancer cells, including the loss of apoptotic control and the ability to migrate into and invade the surrounding tissue, result in cancer cell metastasis to other parts of the body. Therefore, the inhibition of the proliferation, migration, and invasion of cancer cells are the principal goals in the treatment of cancer. This study aimed to investigate the inhibitory activity of nordentatin, a coumarin derivative isolated from *Clausena harmandiana*, regarding the proliferation and migration of human neuroblastoma cells (SH-SY5Y). Nordentatin at a concentration of 100 µM showed cell cytotoxicity toward SH-SY5Y that was significantly different from that of the control group (*p* < 0.01) at 24, 48, and 72 h. Moreover, nordentatin inhibited SH-SY5Y proliferation by inhibiting the antiapoptotic protein Mcl-1, leading to the cleavage of caspase-3 and resulting in the inhibition of a migratory protein, MMP-9, through the GSK-3 pathway (compared with cells treated with a GSK inhibitor). These results suggest that nordentatin inhibited the proliferation and migration of neuroblastoma cells through the GSK-3 pathway.

## 1. Introduction

Cancer is one of the most common diseases in Thailand and throughout the world. As a result of environmental variables and shifting social factors, the incidence rate tends to rise every year. Cancer is also one of the world’s top causes of death. According to the World Health Organization, there were 12.7 million new cases in 2008 and cancer caused the deaths of around 7.6 million people. In Thailand, nearly 80,000 individuals died of cancer in 2018. It is the country’s biggest cause of death. The hallmarks of cancer are unregulated cell division, proliferation, the invasion of neighboring cells, and metastasis in many regions of the body, resulting in tissue death.

Apoptosis, or programmed cell death, is a process that naturally occurs in cells and plays an important role in maintaining the cell balance in higher species. Apoptosis is triggered via two pathways [1]: the extrinsic pathway, or the death receptor pathway, which is triggered by extracellular ligands binding to death receptors, and the intrinsic pathway or mitochondrial pathway, which inhibits abnormal cell division and promotes cell death. Potent molecules that trigger apoptosis in cells are being researched for potential development as anticancer drugs.

Neuroblastoma is the common solid neoplasm of childhood, and its proportion of all pediatric oncology deaths is up to 15%. At the time of diagnosis, approximately 50% of neuroblastoma patients had metastatic locations (clinical stage 4), such as the bone marrow, bone, and liver. Genetic alterations have been shown to be related to neuroblastoma susceptibility. The polymorphism of *ERCC1/XPF* [2] genes encoding for proteins that are involved in DNA repair processes and *LIN28A* [3], which is important in the control of cell proliferation, were associated with increased neuroblastoma risk, and FABP4 [4]-mediated macrophages may increase the characteristics of proliferation and migration in neuroblastoma cells by promoting tumor progression by indirectly altering angiogenesis, matrix metalloproteinases (MMP) activity, and cytokine production [5]. Glycogen synthase kinase-3 (GSK-3) is a critical serine (S)/threonine (T) kinase that regulates several cellular signaling pathways, including cell differentiation, migration, and apoptosis. In cancer, GSK-3 could play a role as a tumor promoter or, in some cases, a tumor suppressor [6]. GSK-3 could regulate NF-κB activity, which is overexpressed in cancer cells. GSK-3 is involved in NF-kB activation and the induction of MMP-9 in cultured rat primary astrocytes [7]. Moreover, GSK-3 could control Mcl-1 stability, which is an important mechanism for the regulation of apoptosis by growth factors, PI3K, and AKT [8].

*Clausena harmandiana*, known as Song-fa in Thai, is a medicinal herb widely found across the northeast part of Thailand. Song-fa was reported in folk medicine textbooks to be prepared as a traditional treatment by boiling it in drinking water. It is said to relieve flatulence, indigestion, fever, and headaches. According to research, the root of Song-fa contains pharmacologically active carbazole alkaloids and coumarins [9,10,11]. It has been shown to have antimalarial [12], antioxidant [13], and antifungal activity [14]. Nordentatin and 7-methoxyheptaphylline were discovered as a carbazole alkaloid and coumarin derivative, respectively, in Song-fa root bark extract. This extract has the ability to trigger apoptosis in intestinal cancer cells (HT-29), increasing the effect of TRAIL on colon cancer cell (HT- 29) death [15]. Therefore, the purpose of this study was to determine the molecular mechanism through which nordentatin (C_19_H_20_O_4_) extracted from Song-fa inhibits cancer cell proliferation. It is believed that the findings of this study will provide vital scientific knowledge for the development of future cancer drugs.

## 2. Materials and Methods

### 2.1. Cell Culture

Eagle’s minimum essential medium (EMEM) containing penicillin, streptomycin, and fetal bovine serum (Gibco BRL, Grand Island, NY, USA) was used to culture SH-SY5Y cells (ATCC) in a T25 cell culture flask at 37 °C in a temperature-controlled incubator with 5% CO_2_. The cell culture medium was changed every 2–3 days.

### 2.2. Study of Inhibitory Activity on Cell Proliferation

To investigate the cell viability, the cells were cultured in 96-well plates with 3 × 10^4^ cells per well and incubated at 37 °C with 5% CO_2_ for 48 h. Nordentatin was dissolved in dimethyl sulfoxide (DMSO) at concentrations of 1, 10, and 100 µM, while doxorubicin (a positive control) was dissolved in DMSO at 10 µM. The chemical treatment was then added to 96-well plates and cultured with the cells for 24, 48, and 72 h periods in a temperature-controlled incubator. The survival of the cells was then determined by removing the test substance, adding 100 µL of MTT reagent (0.5 mg/mL) (Sigma-Aldrich; Merck KGaA) [16], and incubating the cells in a temperature-controlled incubator for 2 to 4 h. The surviving cells reduced the yellow-colored MTT to the dark blue product of formazan MTT. After that, 100 µL of DMSO was added to each well to dissolve the cell precipitate and the absorbance at a wavelength of 570 nm was measured to calculate the percentage of cell viability.

$$\%\;cell\;viability=\frac{Absorbance\;of\;treated\;cells}{Absorbance\;of\;controlled\;\left(untreaed\right)\;cells}\times 100$$

### 2.3. Study of Cell Morphology Change by Phase-Contrast Microscopy

Two milliliters of the cell culture solution were sieved into each well of a 6-well plate with 1 × 10^6^ cells/mL and incubated in a temperature-controlled incubator for a 24 h period. The test substance was added and the plates were incubated in the original state for 24, 48, and 72 h. The cell morphology was observed and photographed using a phase-contrast microscope.

### 2.4. Study of Inhibitory Activity on Cell Migration

Two milliliters of the cell culture solution were added to each well of a 6-well plate with 1 × 10^5^ cells/mL and incubation in a temperature-controlled incubator for a 24 h period. The cells were then treated with the desired active substance at concentrations of 0, 1, 10, and 100 µM or doxorubicin at a concentration of 10 µM for 4 h. The pellets were harvested for each concentration after centrifugation and dissolved in 1 µL of incomplete EMEM. Subsequently, 500 µL of the cell solution was aspirated into a chamber (pore size, 8-mm; Corning Life Sciences). The contents of the chamber were transferred into a well containing 700 µL of complete media (EMEM) and incubated for 24 h. At this time, the medium was removed with a pipette and the cells were fixed with 800 µL of 25% MeOH in each chamber and left for 30 min. The MeOH was withdrawn from the chamber at the end of this time and the chamber was cleaned once with distilled water and twice with PBS. The chambers were allowed to dry upside down and examined for moving cell migration under a microscope.

### 2.5. Cell Lysate Preparation

The cells were incubated with doxorubicin as a control and nordentatin at concentrations of 1, 10, and 100 µM for 4 h. The wells were rinsed with PBS at 4 °C. Lysis buffer (Gibco; Thermo Fisher Scientific, Inc.) was added at 4 °C and 100 µL per well, the wells were scraped, and the contents were aspirated into Eppendorf tubes. The cells were centrifuged for 10 min at 4 °C and 13,500 rpm. After centrifugation, 10 µL of the supernatant was transferred to a 96-well plate. Each well was filled with 190 µL of Bradford’s solution and the plate was shaken. The protein content was measured according to the absorbance at 600 nm to construct standard curves and quantify the protein concentration. The protein concentration was calculated and adjusted using the sample buffer.

### 2.6. Study of the Molecular Mechanism of Nordentatin’s Effect on Cell Apoptosis

The cell lysate was isolated using a protein separation technique, i.e., sodium dodecyl sulfate–polyacrylamide gel electrophoresis (SDS–PAGE) at concentrations of 10 and 12% SDS. The proteins were then transferred from the gel to the membrane using a protein transfer apparatus. The electrophoresis parameters were 0.8 mAmp, 30 W, and 300 V for 90 min. The membrane was removed and placed in a blocking buffer, after which 1 mL of the primary antibody (GSK-3, Mcl-1, Bcl-2, Bcl-xL, cleaved caspase-3, and Actin) (Cell Signaling Technology, Inc.) was added, and the membrane and antibody were shaken at 280 rpm for 2 h. The membrane was washed three times with the washing buffer for 10 min. Next, 1 mL of the secondary antibody was added; the antibody concentration was 0.5:1000 for rabbit and 0.35:1000 for goat. The membrane and antibody were then shaken at 280 rpm for 45 min, and the membrane was washed three times with washing buffer for 10 min. Finally, 300 µL of ECL (Life Science Technology) prepared from a 1:1 luminol enhancer solution + peroxide solution was added. The proteins on the membrane were examined by splicing the film for protein band chemiluminescent detection.

## 3. Results

### 3.1. Study of Inhibitory Activity on Cell Proliferation: Cell Cytotoxicity Assay

**Figure 1:**
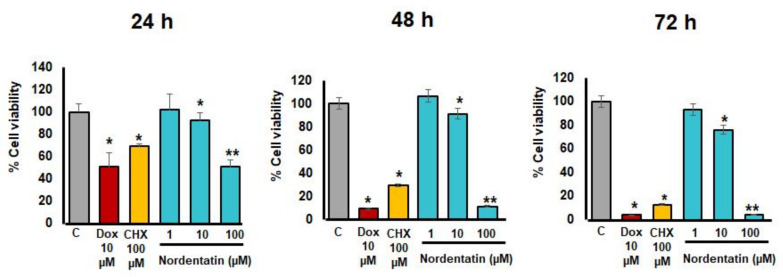
The percentage of neuroblastoma (SH-SY5Y) cells that survived after being exposed to the nordentatin at various concentrations for 24, 48, and 72 h, compared to cells exposed to standardized doxorubicin (Dox) and cycloheximide (CHX). The experiments were conducted in triplicate. * *p* < 0.05, ** *p* < 0.01 versus control group.

The action of a coumarin derivative in Song-fa root bark extract, nordentatin [12], was investigated at various doses. The cytotoxicity was evaluated using the MTT assay and by measuring the absorbance at a wavelength of 570 nm to compute the percentage of cell viability (percent cell viability) (Figure 1).

After 24, 48, and 72 h of incubation, nordentatin at a concentration of 100 µM produced a statistically significant decrease in the percentage of surviving neuroblastoma (SH-SY5Y) cells compared to the control group (*p* < 0.01). Furthermore, at the concentration of 100 µM, the reductions in the percentage of cell survival at 48 and 72 h intervals were significantly different from those at 24 h intervals (*p* < 0.01). The positive controls were doxorubicin and cycloheximide. When comparing the percentage of cell growth inhibition (percent inhibition), 100 µM of nordentatin resulted in 49.21%, 88.49%, and 95.48% inhibition at 24, 48, and 72 h, respectively, while doxorubicin resulted in 49.32%, 90.68%, and 95.72% inhibition at 24, 48, and 72 h.

### 3.2. Study of Cell Morphology Change by Phase-Contrast Microscopy

**Figure 2:**
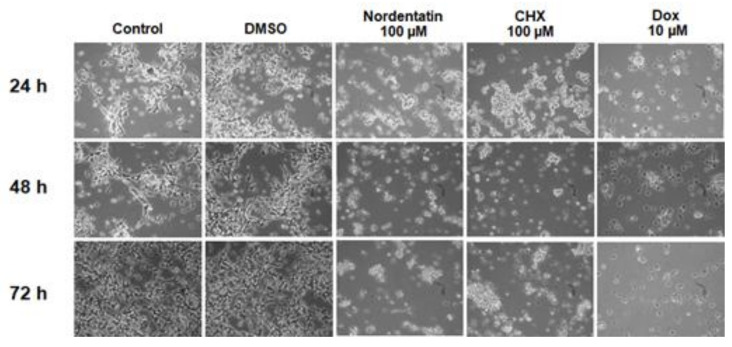
The morphology of SH-SY5Y neural cancer cells treated with chemicals derived from Song-fa as under a phase-contrast microscope.

To determine the type of neuroblastoma cell death that occurred as a result of treatment, the morphological changes of cells treated with nordentatin were investigated using inverted phase-contrast microscopy. Figure 2 depicts the outcomes of the tests.

SH-SY5Y cells were morphologically evaluated to determine the activity of nordentatin. The cell morphology was captured using an inverted phase-contrast microscope. The results show that the morphology of SH-SY5Y cells exposed to 100 µM nordentatin for 24, 48, and 72 h showed distinct apoptosis compared to the control group. As a result, the cancer cells atrophied, becoming smaller and rounder in shape. There was no adhesion to the culture container and the cells floated. SH-SY5Y cells treated with 10 µM doxorubicin and 100 µM cycloheximide for 24, 48, and 72 h showed apoptotic features in their morphology. A large group of cells was separated from a smaller group of cells. The number of cells decreased.

### 3.3. Study of Inhibitory Activity on Cell Migration

**Figure 3:**
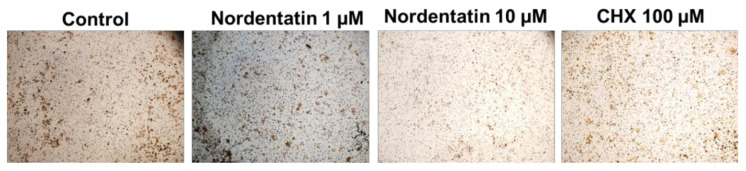
A transwell migration assay was used to investigate the inhibition of neuroblastoma cells (SH-SY5Y).

**Figure 4:**
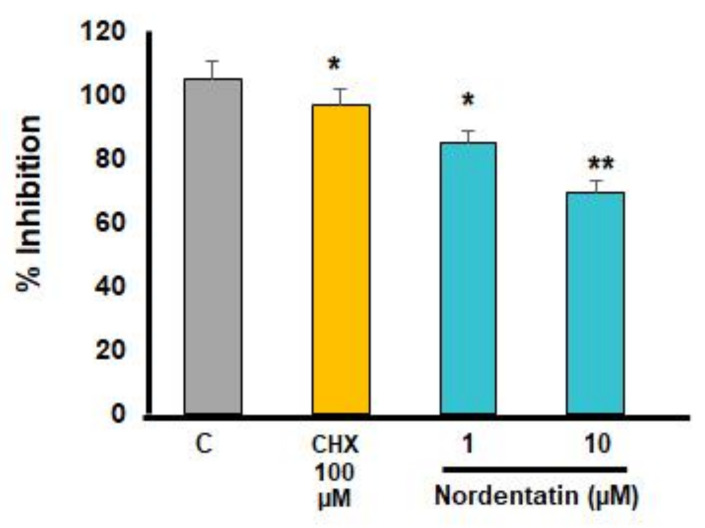
The percentage of neuroblastoma cells (SH-SY5Y) that were able to cross the membrane. The experiments were conducted in triplicate. * *p* < 0.05, ** *p* < 0.01 versus control group.

A transwell migration assay, which is associated with cancer cell metastasis, was used to investigate the effect of nordentatin on the movement and invasion of neuroblastoma cells (SH-SY5Y) (Figure 3 and Figure 4).

There was a reduction in the number of SH-SY5Y cells capable of transwell translocation and hematoxylin staining at 24 h of treatment with nordentatin at 1 µM compared to that in the control group treated with cycloheximide as a positive control. Furthermore, the number of stained cells that decreased in the group treated with 10 µM nordentatin was higher compared to that in the 1 µM group. Nordentatin also had an inhibitory effect on the transwell migration. A cell counter was used to count the number of hematoxylin-stained cells. The number of SH-SY5Y neuroblastoma cells that could pass through the transwell was found to be substantially different (*p* < 0.01) to the positive control treated with cycloheximide. In comparison to the number of neuroblastoma cells (SH-SY5Y) in the transwell, the group treated with nordentatin at 1 µM had a mean of 84 cells, whereas the group treated with nordentatin at 10 µM had a mean of 69 cells, and the cycloheximide treatment showed 97 transwell cells on average. Thus, it was shown that nordentatin inhibited neuronal cell motility (SH-SY5Y) in direct proportion to the concentration.

### 3.4. Study of Molecular Mechanism of Nordentatin’s Effect on Cell Apoptosis

**Figure 5:**
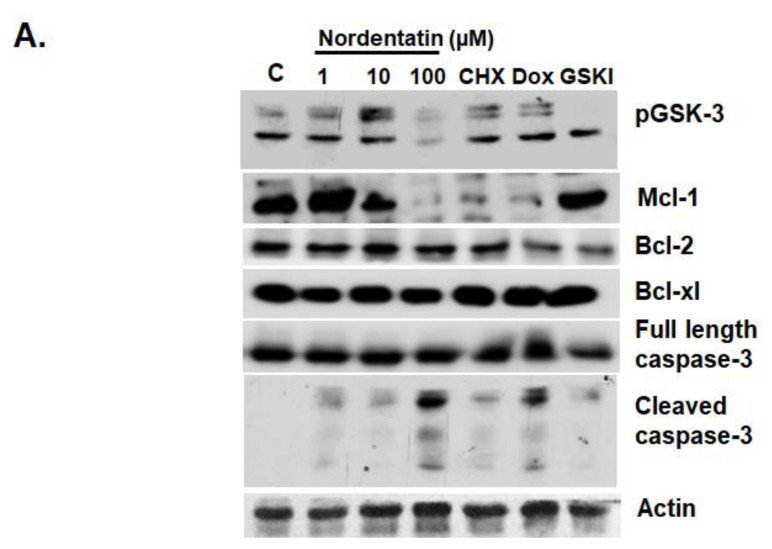

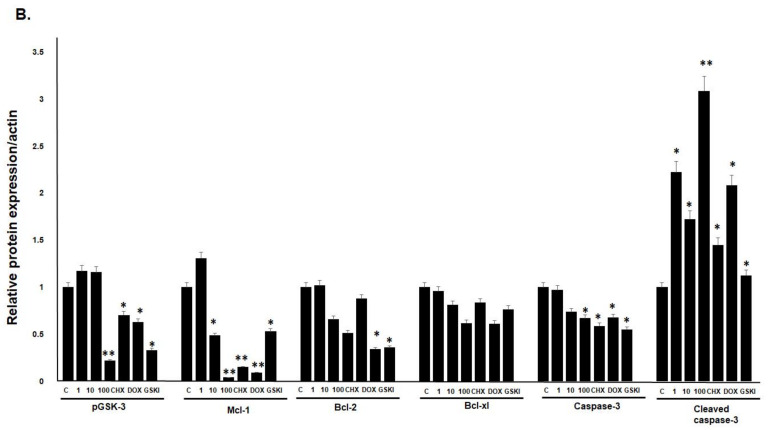
The effects of nordentatin on proteins involved in cell development and apoptosis. (**A**) SH-SY5Y cells were treated with various concentrations of nordentatin, cycloheximide, and doxorubicin for 4 h and compared with the GSK-3 inhibitor. Cell lysates were separated by SDS-PAGE and detected by western blotting using anti-pGSK-3, Mcl-1, Bcl-2, Bcl-xl caspase-3, cleaved caspase-3, and β-actin antibodies. (**B**) Relative phospho-GSK-3, Mcl-1, Bcl-2, Bcl-xl, caspase-3, and cleaved caspase-3 were normalized to that of β-actin, respectively. The experiments were conducted in triplicate. * *p* < 0.05, ** *p* < 0.01 versus control group.

According to studies of cell morphology using phase-contrast microscopy, neuroblastoma cell death occurs in a fashion similar to apoptosis. As a result, the focus of this research was on the molecular pathways that needed to be investigated, including the proteins involved in apoptosis via the intrinsic pathway, with a specific focus on the glycogen synthase kinase-3 pathway (GSK-3 pathway) (Figure 5). A 4 h protein assay was used to study the molecular mechanisms of the action of nordentatin. The protein cysteine–aspartic protease-3 (caspase-3), which is involved in the apoptosis process, was detected via Western blotting. When compared to the control group, cleaved caspase-3 was most clearly exhibited in the 100 µM nordentatin row. Anti-apoptotic proteins, such as myeloid cell leukemia 1 (Mcl-1), were reduced in expression, whereas the B-cell lymphoma-extra-large (Bcl-xl) and B-cell lymphoma 2 (Bcl-2) proteins showed no change.

**Figure 6:**
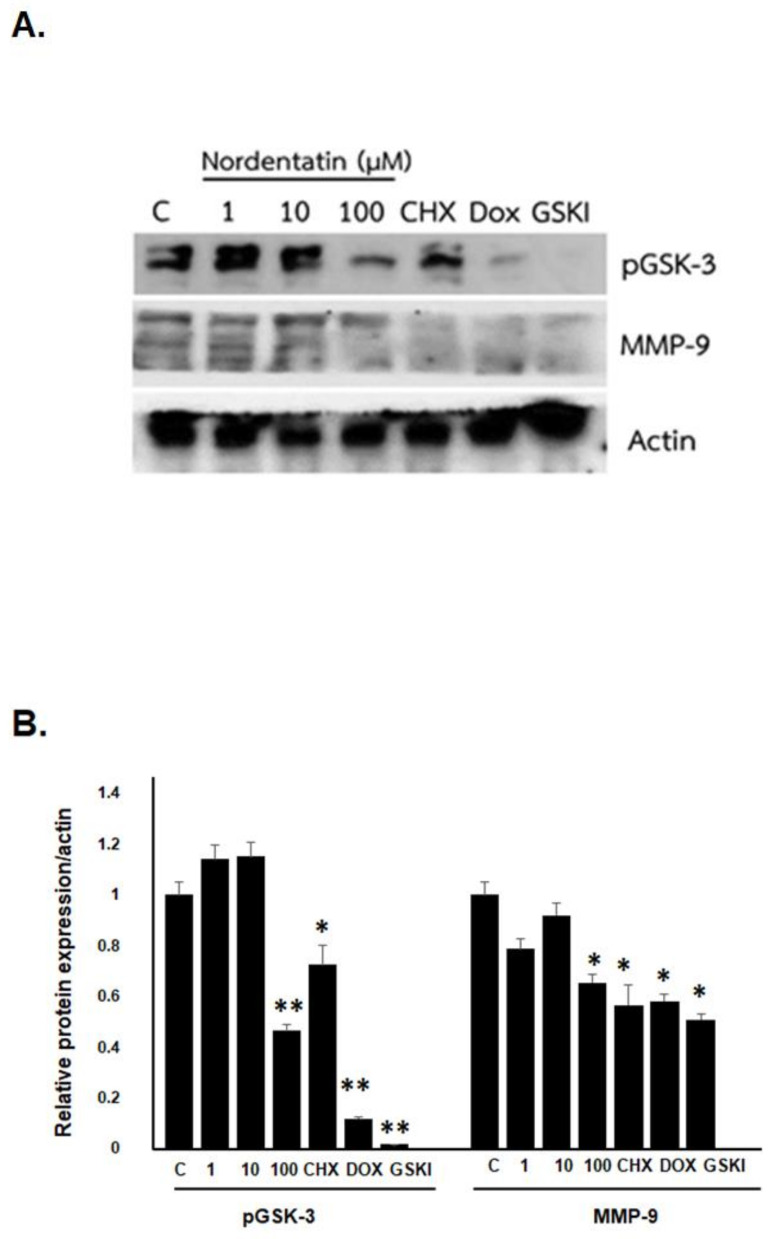
The effect of nordentatin on proteins involved in cell migration. (**A**) SH-SY5Y cells were treated with various concentrations of nordentatin, cycloheximide, and doxorubicin for 4 h and compared with the GSK-3 inhibitor. Cell lysates were separated by SDS–PAGE and detected by western blotting using anti-pGSK-3, MMP-9, and β-actin antibodies. (**B**) Relative phospho-GSK-3 and MMP-9 were normalized to that of β-actin. The experiments were conducted in triplicate. * *p* < 0.05, ** *p* < 0.01 versus control group.

Matrix metalloprotein-9 (MMP-9) is a proteinase and collagenase that can break down extracellular matrix components, allowing cells to migrate and invade. The effect of nordentatin on MMP-9 expression and its implications in neuroblastoma migration and invasion were investigated. In a 10 min test, it was discovered that the expression of the MMP-9 protein in the 100 µM nordentatin row was much lower than that in the control group. Furthermore, at 100 µM, glycogen synthase kinase-3 (GSK-3)’s protein expression began to drop in the nordentatin row compared to the control group. In addition, GSK-3 expression was shown to be significantly reduced in the GSK inhibitor line. Moreover, the expression of MMP-9 was reduced, implying that nordentatin prevented GSK-3 expression (Figure 6), thereby reducing the expression of the MMP-9 protein and thus limiting cancer cell growth.

## 4. Discussion

Neuroblastoma, a pediatric cancer of the peripheral sympathetic nervous system, is frequently diagnosed at the advanced stage of disease and treated with various strategies, including chemotherapy, radiotherapy, autologous stem cell transplantation, and surgery [15,17]. Despite several treatment strategies being effective, the majority of patients relapse with metastatic disease [18,19,20]. Novel therapies for neuroblastoma are urgently required. Agents able to decrease cell proliferation, induce apoptosis, or inhibit metastasis (migration and invasion) are currently used for the treatment of cancer. Agents with multiple targets are considered to be more effective than a single-target agent for complex diseases, especially cancer [16].

Several studies have introduced coumarins as potential multi-target agents with a wide spectrum of pharmacological effects [21,22]. Coumarins, naturally occurring phytochemicals, have been reported as promising anticancer compounds against several types of cancer via different mechanisms [23,24,25,26]. Among coumarins, nordentatin isolated from the root bark of *Clausena harmandiana* showed high cytotoxic activity. A recent study showed that nordentatin possesses potential anti-tumor activity in the small lung cancer (NCI-H187), breast cancer (MCF-7), and oral cavity cancer (KB) cell lines [27]. However, in the reviewed literature, the effect of nordentatin on neuroblastoma has not been investigated. In the present study, the antitumor effect and underlying mechanisms of nordentatin isolated from the root bark of *Clausena harmandiana* were investigated in neuroblastoma cells. This study focused on the induction of apoptosis and its effect on the proliferation and invasion of neuroblastoma cells.

Apoptosis is a programmed cell death regulated by the Bcl-2 family and caspase cascade, including caspase-9, caspase-3, and caspase-7 [28]. Anti-apoptotic Bcl-2 family proteins, usually over-expressed in human cancers, have been reported to play an important role in the resistance to chemotherapeutic drugs. The Bcl-2 family protein consists of anti-apoptotic proteins (Mcl-1, Bcl-xL and Bcl-2) and pro-apoptotic proteins that include the BH3-only proteins, including Bim, Bad, Bid, Noxa, and Puma, and the multi-domain molecules Bax and Bak [29]. After the activation of Bax and Bak by apoptosis stimuli, cytochrome *c* is released from the mitochondria to the cytoplasm and binds to apoptotic protease-activating factor-1 (APAF-1) and caspase-9 to form apoptosome, which is subsequence to the activation of the caspase cascade [30]. Anti-apoptotic proteins are able to stabilize Bax and Bak activation and protect cancer cells from apoptosis stimuli [31]. A cytotoxicity study was conducted to investigate nordentatin inducing the apoptosis of neuroblastoma cells. Human neuroblastoma cells (SH-SY5Y) were incubated with gradient doses of nordentatin at concentrations of 1, 10, and 100 µM for 24, 48, and 72 h, and an MTT assay was performed. Our results demonstrated that nordentatin was able to reduce cell viability and induce changes in cell morphology. The MTT assay revealed that nordentatin reduced the viability of SH-SY5Y cells in a dose-dependent manner. Interestingly, nordentatin at a concentration of 100 μM showed a better effect than the cycloheximide used as a positive control. Our result indicates that nordentatin has a cytotoxic impact on cancer cells.

We next used inverted phase-contrast microscopy to examine the morphological changes in cells treated with nordentatin. The morphological investigation of SH-SY5Y cells treated with 100 µM nordentatin for 24 h revealed substantially more cell death features than the control group. There was the shedding of cells from the adhesion surface and the shrinkage of cells. Such death is similar to that which occurs in apoptosis. The results indicate that nordentatin induces cancer cell death through an apoptotic pathway. Our results showed that nordentatin induced increased levels of cleaved caspase-3, a marker of apoptosis. Nordentatin reduced the expression of Mcl-1 proteins in SH-SY5Y cells, but had no effect on the expression of Bcl-2 and Bcl-xl proteins, which were typically responsible for preventing apoptosis.

Matrix metalloproteinases (MMPs) are proteolytic metalloenzymes that play a role in the degradation of a number of extracellular matrix (ECM) components. MMP-9, also known as gelatinase B, is a matrix metalloproteinase with the most complex form. The overexpression and dysregulation of MMP-9 are associated with migration and invasion in cancer. The regulation and inhibition of MMP-9 may inhibit cancer metastasis. The discovery of a new selective MMP-9 inhibitor may be useful in cancer prevention and treatment in the future. It has been reported that the siRNA-mediated knockdown of MMP-9, uPAR, and CB inhibits the invasiveness and migration of prostate cancer cells and leads to apoptosis, both in vitro and in vivo [32]. α-Hispanolol induced apoptosis and inhibits the cell migration of glioblastoma cells by a decreased level of MMP-9 [33]. The study of the inhibition of the migration and invasion of neuroblastoma cells by a transwell migration assay showed that nordentatin has the ability to inhibit the motility and invasion of nerve cancer cells. Regarding the effect of nordentatin on the protein matrix metalloprotein-9 (MMP-9), which is a proteinase and collagenase, the results indicated that there was clear suppression of MMP-9 expression in the nordentatin test compared to the control group.

GSK-3 is a regulator of NF-κB, which is expressed in cancer and immunological disorders [34,35]. According to the findings of Ding et al. [36], Inuzuka et al. [37], Maurer et al. [8], Morel et al. [38], and Zhao et al. [39], Mcl-1 expression is controlled by the GSK-3 protein and Mcl-1 is lost through stabilization. It has also been observed to increase the production of cleaved caspase-3, which could induce apoptosis [40]. Kitano et al. [41] discovered that inhibiting GSK-3 expression could decrease cancer cell motility and invasion. The inhibition of GSK-3 regulates MMP-9 expression in various tissues [42]. It has been shown that the GSK-3 inhibitor SB216763 induces apoptosis by suppressing the NF-κB signaling pathway in osteosarcoma cells [43]. The GSK-3β inhibitor AZD1080 suppresses ovarian cancer cell proliferation, invasion, and migration and downregulates GSK-3β, MMP9, and Bcl-xL mRNA and proteins [44]. In this study, we investigated the proteins involved in apoptosis via the intrinsic pathway, with a particular emphasis on the GSK-3 pathway. We found that nordentatin inhibited the phosphorylation of GSK-3 and Mcl-1 expression, resulting in the activation of caspase-3 and apoptosis. Furthermore, GSK-3 phosphorylation was reduced in the nordentatin-treated group, correlated with a decrease in the MMP-9 level. Therefore, nordentatin can be inferred to impede the proliferation and migration of neural cancer cells via the GSK-3 pathway.

## 5. Conclusions

**Figure 7:**
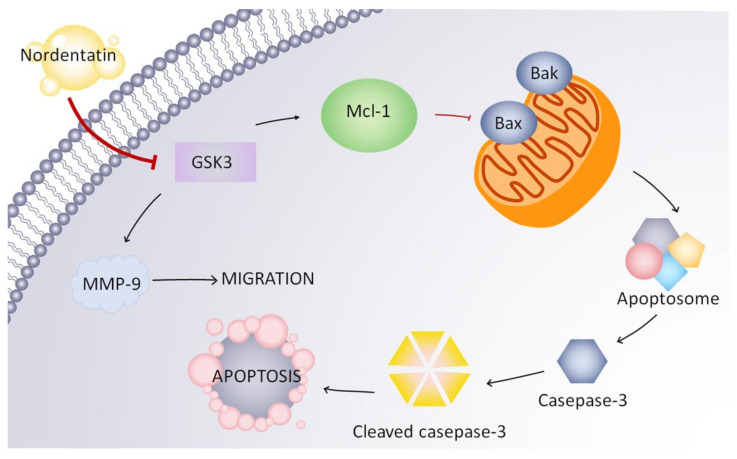
The effect of nordentatin on the GSK-3 pathway in the apoptosis and migration of SH-SY5Y cells.

In conclusion, this study investigated the effect of nordentatin from *Clausena harmandiana* on SH-SY5Y cancer cell proliferation and migration. It was found that nordentatin inhibited the proliferation and migration of SH-SY5Y cells by inhibiting GSK-3 phosphorylation, which controls the expression of Mcl-1 (antiapoptotic protein), resulting in the activation of cleaved caspase-3 and the apoptosis of cancer cells. Moreover, the inhibition of nordentatin on GSK-3 also suppressed the expression of MMP-9, which regulated cell migration (Figure 7). This study indicated that nordentatin inhibited SHSY5Y cell proliferation and migration through the GSK-3 pathway. Thus, nordentatin could be considered in the future as a lead for further drug synthesis or, due to it being a medicinal plant constituent, as a chemopreventive agent.

## Data Availability

The data presented in this study are available on request from the corresponding authors.
